# Maternal nutritional manipulations program adipose tissue dysfunction in offspring

**DOI:** 10.3389/fphys.2015.00158

**Published:** 2015-05-13

**Authors:** Simon Lecoutre, Christophe Breton

**Affiliations:** Unité Environnement Périnatal et Santé, UPRES EA 4489, Equipe Malnutrition Maternelle et Programmation des Maladies Métaboliques, Université de LilleVilleneuve d'Ascq, France

**Keywords:** adipocyte, hypertrophy, hyperplasia, fetal programming, epigenetic mechanisms, inflammation, obesity

## Abstract

Based on the concept of Developmental Origin of Health and Disease, both human and animal studies have demonstrated a close link between nutrient supply perturbations in the fetus or neonate (i.e., maternal undernutrition, obesity, gestational diabetes and/or rapid catch-up growth) and increased risk of adult-onset obesity. Indeed, the adipose tissue has been recognized as a key target of developmental programming in a sex-and depot-specific manner. Despite different developmental time windows, similar mechanisms of adipose tissue programming have been described in rodents and in bigger mammals (sheep, primates). Maternal nutritional manipulations reprogram offspring's adipose tissue resulting in series of alterations: enhanced adipogenesis and lipogenesis, impaired sympathetic activity with reduced noradrenergic innervations and thermogenesis as well as low-grade inflammation. These changes affect adipose tissue development, distribution and composition predisposing offspring to fat accumulation. Modifications of hormonal tissue sensitivity (i.e., leptin, insulin, glucocorticoids) and/or epigenetic mechanisms leading to persistent changes in gene expression may account for long-lasting programming across generations.

Epidemiological and clinical studies demonstrated that nutrient supply perturbations in the fetus or neonate (i.e., maternal undernutrition, obesity, gestational diabetes and/or rapid catch-up growth) are associated with higher adiposity in adulthood (Ravelli et al., 1999). Based on the concept of Developmental Origin of Health and Disease (Barker, 2004), it has been hypothesized that maternal nutritional manipulations during the perinatal period may program the development and cause dysfunction in offspring's adipose tissue later in life. Over the past decade, an increasing number of data from human and animal studies have validated this concept (Lukaszewski et al., 2013; Lecoutre and Breton, 2014).

Several types of adipose tissue coexist in mammals. Although their properties are quite different, they exhibit similar cell composition: mature adipocytes (i.e., lipid storage compartment) and a stromal vascular fraction (a heterogeneous population of cell types including adipose stem cells). The white adipose tissue (WAT) constitutes the main energy reserve, storing triglycerides (TG) during periods of positive energy balance by promoting lipogenesis. Two distinct processes account for WAT growth. First, adipogenesis (i.e., increased adipocyte number) relies on the recruitment and the commitment of adipose stem cells to adipocyte lineage. The differentiation of preadipocytes into adipocytes are regulated by several adipogenic and lipogenic transcription factors such as peroxisome proliferator-activated receptor-γ (PPARγ), CCAAT/enhancer binding protein (C/EBPα, β, γ), the sterol regulatory element-binding protein 1c (SREBP1c) as well as fatty acid synthesis enzymes such as fatty acid synthase (FAS). Second, lipogenesis (i.e., increased adipocyte size) depends on the synthesis and the storage of TG in mature adipocyte (Figure 1). Fat cell number and lipolysis are also controlled by the activity of the WAT sympathetic system (Bowers et al., 2004). The brown adipose tissue (BAT) differs from WAT by its cell origin (Seale et al., 2009). Brown adipocytes dissipate energy via thermogenesis mediated via BAT-selective genes such as uncoupling protein 1 (UCP1) and transcription factors [PPARα and PPARγ coactivator 1-α (PGC1-α)]. Despite high similarities to brown adipocytes, brite adipocytes (brown-in-white) derive from a distinct origin and are closer to the white adipocyte cell lineage. Several studies in rodents showed that WAT browning (i.e., enhanced brite adipocytes) can be induced by prolonged cold exposure, treatment with β-adrenergic agonists (via β3-adrenoreceptor activation) and endurance exercise (Vosselman et al., 2013).

**Figure 1:**
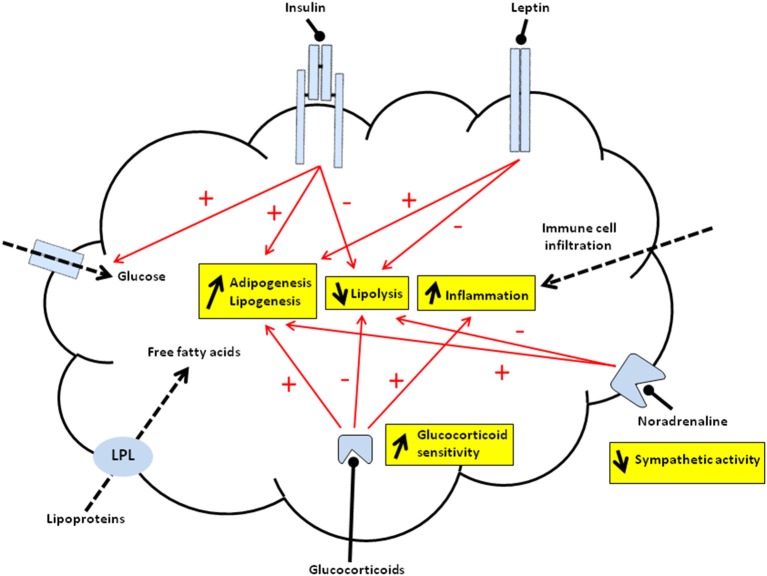
**Schematic representation of programmed mechanisms in offspring's adipose tissue of malnourished dams**. Overall, offspring from malnourished dams display increased adipogenesis/lipogenesis and inflammation as well as decreased lipolysis within adipose tissue. Red arrows indicate programmed activation (+) or inhibition (−) of major processes controlled by hormones that may predispose offspring to fat accumulation. The accumulation of triglycerides (TG) may be due to increased esterification of either free fatty acids released from lipoproteins, catalyzed by lipoprotein lipase (LPL) (dashed arrow), or free fatty acids synthesized from glucose metabolism mainly driven by lipogenic enzymes. Leptin acting via its receptor (JAK2/STAT3 signaling pathway) may activate preadipocyte proliferation and/or inhibit lipolysis. Insulin after binding to its receptor (IRS/PI3K/Akt signaling pathway) may promote the TG formation via activation of glucose entry (i.e., increased GLUT4 translocation) (dashed arrow) and lipogenic enzymes. Insulin also shows anti-lipolytic effects and may activate adipogenesis. Increased adipogenesis and decreased lipolysis may be due to impaired activity of the sympathetic system (via noradrenaline). Glucocorticoids (GC) that bind intracellular receptor may activate adipogenesis and lipogenesis whereas they inhibit lipolysis. Thus, both increased circulating GC levels and intracellular GC sensivity (i.e., modified GR, MR, 11 β-HSD1, 11 β-HSD2 relative abundance and activities) can directly impact on adipose tissue function. The pro-inflammatory state might be due to either immune cell infiltration (dashed arrow) and/or local expression of pro-inflammatory mediators. The pro-inflammatory state may also originate, at least in part, from increased GC sensitivity within WAT.

Because adipose tissue development occurs at different periods during the perinatal period in rodents (i.e., mainly at the end of gestation, throughout lactation and adolescence) and in bigger mammals (i.e., before birth), the window of vulnerability to environmental factors differs between species (Muhlhausler and Smith, 2009; de Oliveira et al., 2013). However, regardless of the difference in the timing of adipogenesis and adipose tissue development, closely related mechanisms underlying perinatal programming have been reported in altricial and precocial species (Lukaszewski et al., 2013; Lecoutre and Breton, 2014). In this context, we provide a brief overview on the repercussion of perinatal nutritional manipulations on adipose tissue functionality in programmed offspring.

## Maternal nutritional manipulations alter adipogenesis and lipogenesis in offspring's adipose tissue

### Low birth-weight offspring

Three principal models of maternal undernutrition leading to low birth weight have been developed: maternal low-protein diet (LP), maternal food restriction (FR) and uterine artery ligation (mimicking uteroplacental insufficiency) in pregnant dams.

Maternal LP during gestation and lactation results in a reduction in adipose cell size of rat offspring (Ferland-McCollough et al., 2012). Analysis of WAT of adult offspring from LP dams revealed an upregulation of C/EBPα and PPARγ gene expression (Guan et al., 2005) with increased rate of cultured preadipocyte proliferation (Bieswal et al., 2004). In addition, adult rats from LP dams displayed elevated miRNA-483-3p expression levels that may reduce the capacity of lipid storage contributing to limit adipocyte hypertrophy (Ferland-McCollough et al., 2012). In contrast, rat offspring from 50% food-restricted (FR50) dams during gestation from day 10 to term (Desai et al., 2008) or from 70% food-restricted (FR70) dams throughout the gestation (Lukaszewski et al., 2011) showed hypertrophic adipocytes. Newborn rats from FR50 dams displayed higher preadipocyte proliferation, C/EBPs and PPARγ expression, along with adipocyte TG accumulation *in vitro* (Yee et al., 2012). Prior to the onset of overt obesity, increased adipocyte size was associated with elevated expression levels of factors promoting lipogenesis (i.e., SREBP1c, FAS, leptin) in weanling pups (Desai et al., 2008) and adult offspring (Lukaszewski et al., 2011) from FR dams. Similarly, uterine artery ligation of pregnant dams causes an upregulation of PPARγ gene expression in WAT of rat offspring before increased adiposity (Joss-Moore et al., 2010).

Several animal model and human studies have highlighted the deleterious effect of rapid postnatal growth, especially when overfeeding paradigms during lactation (i.e., hypercaloric maternal diet, cross-fostering pups, reduced litter size) were applied in low-birth-weight offspring. In particular, the mismatch between pre- and postnatal energy supply was closely related to increased adipogenesis and higher risk of obesity later in life (Druet et al., 2012). Consistent with these findings, postnatal overfeeding of rat pups from LP dams promoted higher proliferation and differentiation rate of cultured preadipocytes (Bol et al., 2008). Overfed adult offspring from FR dams exhibited higher adiposity along with increased lipogenic (Lukaszewski et al., 2011) and clock genes (Sutton et al., 2010) in a depot-specific manner. The latter suggests that circadian rhythm disruptions may participate to the adult-onset obesity. Similar patterns of programming are observed in sheep, which show great similarities with the development of human adipose tissue. Low birth-weight lamb with atrophied fat depots displayed progressive fat accumulation along with increased PPARγ gene expression through accelerated postnatal growth (Muhlhausler and Smith, 2009).

### Maternal overfeeding

Numerous models of maternal overfeeding and obesity [i.e., high-fat (HF) or cafeteria diet] before and/or during gestation and/or lactation were described in the literature. In most cases, despite normal birth weight, offspring of obese dams are sensitized to postnatal adiposity, adipocyte hypertrophy and weight gain.

Several rodent and sheep models of maternal obesity demonstrate that maternal obesity at conception enhances adipogenesis from the fetal period (Muhlhausler and Smith, 2009; Borengasser et al., 2013) to adulthood (Murabayashi et al., 2013) resulting in higher WAT mass and larger adipocytes. Overfeeding during lactation and/or postweaning periods leading to catch-up growth, consistently worsen adipogenesis programming (Desai and Ross, 2011; Guberman et al., 2013). Upregulated PPARγ is one of the characteristic feature of enhanced adipogenesis and fat expansion in programmed offspring of obese dams (Samuelsson et al., 2008; Muhlhausler and Smith, 2009). This is associated with downregulated PPARγ corepressors (Desai and Ross, 2011). In addition to higher amount of TG along with lipoprotein lipase (LPL) activity within WAT (Figure 1), obesity prone-rats from cafeteria-diet-fed dams throughout gestation and lactation exhibited modified fatty acid composition (Benkalfat et al., 2011). In rodents, the predisposition of adiposity in offspring following maternal overfeeding occurs in a sex- and species-dependent manner. In accordance with these findings, only female offspring of mice from HF-fed dams throughout gestation and lactation displayed hypertrophied adipocyte with blunted lipolytic capacities (Samuelsson et al., 2008). Using a similar maternal nutritional paradigm, we observed an opposite phenotype in rat offspring (i.e., male obese vs. lean female). Maternal gestational diabetes also predispose adult offspring to adipocyte hypertrophy and obesity (Steculorum and Bouret, 2011), suggesting that modified insulin and/or glucose levels during the perinatal period program adipose dysfunction in offspring (Figure 1).

### Modified energy intake during the suckling period

Given that the lactation coincides with the period of maximum adipogenesis in rodents, the modification of milk intake by rearing pups in small (overnutrition) or large litters (undernutrition) has been used extensively to investigate adipose tissue development.

In rats, neonatal overfeeding (i.e., pups reared in small litters) led to rapid weight gain during lactation that remains visible until adulthood. The perinatal period is characterized by hyperinsulinemia, known to participate to the developmental programming of fat accumulation (Figure 1). During this period, adipose tissue expansion is due both to hyperplasia (i.e., higher preadipocyte and stromal cell numbers) and hypertrophy (i.e., enhanced lipogenesis such as LPL activity) within WAT. In adult offspring, increased adiposity mainly resulted from adipocyte enlargment [i.e., higher lipogenesis and modified sensitivity of glucocorticoid (GC)] in a depot-specific manner (Boullu-Ciocca et al., 2008) (Figure 1). Similar outcomes (i.e., neonatal hyperinsulinemia, hypertrophied adipocytes, exacerbated lipogenesis and obesity later in life) were also associated with artificially reared rat pups fed a formula high in carbohydrate-derived energy. These findings highlight the importance of modified perinatal glucose and/or insulin levels in programming events (Srinivasan et al., 2008). In contrast, both paradigms of neonatal underfeeding in rats (i.e., pups reared in large litters or breastfed by FR30 dams) resulted in lean phenotype with smaller adipocytes and protection to diet-induced obesity (Patterson et al., 2010; Palou et al., 2011).

## Maternal nutritional manipulations impair sympathetic activity in offspring's adipose tissue

We showed that perinatal maternal undernutrition (FR50 model during the last week of gestation and lactation) resulted in a delay in the development of gonadal WAT in male rat at weaning. The neonatal WAT was characterized by the appearance of brown-like adipocytes and increased markers of thermogenesis (i.e., UCP1, PGC1α and PPARα). This phenomenon might, at least in part, rely on exacerbated WAT sympathetic activity recruited in offspring to promote browning, thereby increasing the capacity for adaptive thermogenesis (Delahaye et al., 2010). In agreement with these findings, adult rat male offspring from FR20 dams during the first 12 days of pregnancy, but not females, displayed an increase in adipose tissue cellularity along with alterations of WAT sympathetic innervations (García et al., 2011). Reduced BAT mass and UCP1 activity were also closely related to impaired sympathetic activity and lipolysis in adult rats reared in small litters (Xue et al., 2007).

## Offspring of malnourished dams show increased inflammatory response in adipose tissue

Offspring of malnourished dams displayed chronic low-grade obesity-associated inflammation characterized by elevation of inflammatory factors in plasma (i.e., TNF-α, IL-6 and MCP-1) and expression of pro-inflammatory mediators in WAT. The latter may be due to increased production of cytokines by adipocytes and/or immune cells that infiltrated within WAT. The hallmark of the WAT inflammatory is the appearance prior to overt obesity. Thus, elevated pro-inflammatory markers in WAT, possibly originating from immune cell infiltration were reported in fetus of obese mice fed a cafeteria diet before mating and throughout gestation (Murabayashi et al., 2013). This phenomenon occurred at an early stage of WAT development in line with increased storage of TG in prenatally undernourished adult sheep (Sharkey et al., 2009) and rat offspring following uteroplacental insufficiency (Joss-Moore et al., 2010). Rat offspring reared in small litters (Boullu-Ciocca et al., 2008) as well as juvenile offspring from obese HF-fed rats (Del Bas et al., 2015) also displayed a early postnatal induction of pro-inflammatory cytokine mRNA expression levels in WAT that were exacerbated under HF diet. However, *in utero* HF diet exposure may result in increased pro-inflammatory markers in WAT of adult mice offspring, independently of maternal obesity (Umekawa et al., 2015). The pro-inflammatory state may also originate, at least in part, from modified GC sensitivity within WAT (Lee et al., 2014). However, it remains to determine whether inflammatory changes are cause or consequence of fat accumulation.

## Programming mechanisms

Numerous studies regarding nutritional manipulations in the perinatal period using different opposite paradigms (undernutrition vs. overfeeding) pointed out the fact that redundant mechanisms rely on the programming of adult offspring's adipose tissue. Some of them occur in a gender-specific manner.

## Plasma hormone levels and tissue sensitivity

Over the past few decades, the adipocytokine leptin has been considered as the main programming factor of the hypothalamus adipose-axis. Originally, leptin was described as a hypothalamic neurotrophic factor involved in the plasticity and hardwiring of the appetite regulatory circuits in the hypothalamus (Bouret et al., 2004). Numerous studies indicated that perinatal leptin manipulations result in increased risk of adult-onset obesity (Breton, 2013). Indeed, altered postnatal leptin surge during lactation observed in undernourished (Delahaye et al., 2008; Palou et al., 2012) or overnourished rodent (Kirk et al., 2009) is associated with fat accumulation in adulthood. In addition, early postnatal leptin blockage increases susceptibility to diet induced obesity in rats (Attig et al., 2008) whereas administration of leptin during the postnatal period reverses obesity in prenatally undernourished adult rats (Vickers et al., 2008). Interestingly, leptin may act directly on adipose tissue via binding to its receptor to increase preadipocyte proliferation (Bol et al., 2008) and has differential morphogenesis effects on male and female adipocytes (Guo et al., 2009). It may also inhibit lipogenesis in already developed adipocytes (Huan et al., 2003) (Figure 1). Insulin has been also reported as programming factor on the hypothalamus adipose-axis. Thus, perinatal manipulations of insulin levels (Breton, 2013) and altered insulin signaling in the hypothalamus (Vogt et al., 2014) result in fat accumulation in offspring. Fat cells respond to insulin after binding to its receptor and activation of the PI3K/Akt signaling pathways by activating adipogenesis/lipogenesis and inhibiting lipolysis (Poulos et al., 2010) (Figure 1).

The link between chronic excess of plasma GC levels and adiposity as observed in Cushing's syndrome suggested that altered GC metabolism may also predispose to fat expansion. This observation has led to the hypothesis that perinatal hypercorticosteronemia due to disturbed HPA axis feedback might be a key factor in offspring's WAT programming (Breton, 2013). First, GC alone or in interaction with insulin is known to regulate *in vitro* and *in vivo* the differentiation of adipocyte precursors (i.e. increased C/EBP**α**, PPAR**γ** ) and lipogenic genes (Figure 1). Second, GC may be the source of chronic inflammatory conditions in WAT by inducing inflammation-related gene expression and pro-inflammatory immune cell infiltration (Lee et al., 2014).

In addition to higher systemic GC levels, local induction of GC activity within WAT due to modified GR, MR, 11β-hydroxysteroid dehydrogenase type 1 (11**β**-HSD1), 11β-hydroxysteroid dehydrogenase type 2 (11**β**-HSD2) relative abundance, may also account for increased adiposity (Lee et al., 2014) (Figure 1). Several studies using maternal nutritional manipulation models support this notion. Perinatally undernourished offspring of precocious species (primate, sheep) that are sensitized to weight gain, exhibited increased glucocorticoid receptor (GR) and 11β-HSD1 gene expression, enzyme that regenerates intracellular GC by converting inactive GC metabolites to active GC during WAT development. This phenomenon takes place *in utero* during early fetal development of adipose tissue in female but not male primate (Guo et al., 2013). In low birth weight lambs, the modified GC sensitivity was mainly observed during catch-up growth period along with decreased 11β-HSD2 that modifies active GC to inactive metabolites (Gnanalingham et al., 2005). In rodents, obesity-prone rat reared in small litters and from diabetic dams also exhibited an induction of GR and 11β-HSD1 gene expression in WAT during the postnatal period. The increase in intracellular GC sensitivity paralleled accelerated WAT growth but took place before overt obesity (Boullu-Ciocca et al., 2008). Why postweaning HF diet accentuates the GC sensitivity within WAT remains to be determined.

The action of GC in WAT is far less than clear but mainly depends on local balance of active GC *versus* inactive GC metabolites by 11β-HSD1 and 11β-HSD2 (Lee et al., 2014). In accordance with this notion, we observed a modified 11β-HSD1/11β-HSD2 ratio in adult rat offspring from FR70 dams throughout gestation as well as from HF-fed dams throughout gestation and lactation (Lukaszewski et al., 2011). In particular, the depot-specific decreased 11β-HSD1/11β-HSD2 ratio might be seen as an adaptive mechanism to limit fat accumulation (Lukaszewski et al., 2011).

## Epigenetic and transgenerational mechanisms

Parental nutritional manipulations during critical developmental time windows may permanently modulate gene expression profiles in progeny via epigenetic mechanisms (i.e., DNA methylation, histone modification and non-coding RNA modifications). Thus, the implication of these epigenetic processes have become increasingly important. It might account for increased risk of adult-onset non-communicable diseases such as obesity (Lillycrop and Burdge, 2012). In particular, perinatally unbalanced maternal diet was showed to induce epigenetic changes in offspring genome resulting in long-lasting modification of the transcriptional control of adipogenesis (Musri and Párrizas, 2012) and/or in inflammation (Toubal et al., 2013). An isocaloric LP diet given to dams throughout gestation and lactation affects the CpG site methylation of the leptin promoter, adipose expression and plasma levels in adult offspring mice (Jousse et al., 2011). A similar LP diet applied to rat dams leads to higher miRNA-483-3p levels, known to reduce adipose tissue expandability in rat offspring (Ferland-McCollough et al., 2012). Maternal obesity in female mice induces increased gene expression of Zfp423, a key transcriptional factor initiating adipogenic commitment, along with lower promoter methylation levels in fetal offspring (Yang et al., 2013). Similarly, weanling rats from obese dams display greater adipocyte differentiation, increased Zfp423, PPARγ and C/EBPβ mRNA expression levels along with specific alterations in DNA methylation of CpG sites (Borengasser et al., 2013). Maternal HF diet during pregnancy also leads to histone modifications within leptin and adiponectin promoter regions that may affect adipocytokine gene expression in mouse offspring (Masuyama and Hiramatsu, 2012).

Perinatal perturbations of fetus/neonate nutrient supply program obesity differently according to the sex of the offspring (Samuelsson et al., 2008; García et al., 2011; Guo et al., 2013). The basis of the sex specific programming effects remains elusive but could reflect direct interactions between maternal nutritional signals and mechanisms and/or factors involved in sex differences in development (i.e., differences in patterns and in timing of development and influence of steroid hormone exposure during the perinatal period) (Aiken and Ozanne, 2013). This phenomenon may be due, at least in part, to gender-specific epigenetic modifications during early stages of the development. In agreement with this notion, the global methylation profile in placenta as well as gene expression pattern was obviously different between male and female offspring from HF-fed obese mice (Gallou-Kabani et al., 2010). Changes to the epigenome associated with first generation phenotype can last through subsequent generations to promote transgenerational inheritance via both maternal and paternal lineages. In the first generation, somatic programming may acutely affect tissue, leading to offspring phenotype. At the same time, the germ cell lineage which transmits genetic and epigenetic information from one generation to the next, may undergo epigenome reprogramming during their development (Dunn et al., 2011). Based on the transgenerational inheritance concept, the multigenerational HF-driven obesity may be seen as a vicious circle that may participate to the transmission of obesity. Accordingly, HF-fed female progeny of obese mice dams displayed WAT inflammation and obesity over three generations. Multigenerational changes in DNA hypomethylation on inflammatory genes contribute, at least in part, to these phenomena (Ding et al., 2014). In humans, epidemiological studies using the Dutch famine birth cohort have also highlighted transgenerational transmission of obesity (i.e., increased neonatal adiposity of the second generation offspring from undernourished mothers) (Painter et al., 2008).

Interestingly, maternal diet supplementation (including methyl donors) during gestation and/or lactation might partially prevent the obese phenotype of offspring (Cordero et al., 2014). Although underlying mechanisms remain elusive, epigenetic processes (i.e., modified methylation status) may account for deprogramming events (Lillycrop et al., 2005). Thus, maternal diet supplementation via offspring's epigenome changes may constitute a promising strategy (perhaps even prevention) for early intervention in order to alleviate deleterious programming effects of maternal malnutrition.

### Conflict of interest statement

The authors declare that the research was conducted in the absence of any commercial or financial relationships that could be construed as a potential conflict of interest.
